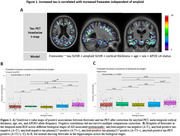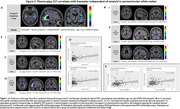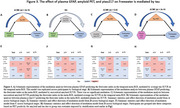# Cortical freewater increases in areas of tau tangle aggregation, independent of cortical thinning

**DOI:** 10.1002/alz70856_105163

**Published:** 2026-01-08

**Authors:** Brandon J Hall, Etienne Aumont, Arnaud Boré, Joseph Therriault, Seyyed Ali Hosseini, Nesrine Rahmouni, Arthur C. Macedo, Stijn Servaes, Gleb Bezgin, Jaime Fernandez Arias, Yi‐Ting Wang, Tevy Chan, Lydia Trudel, Jenna Stevenson, Andrea Benedet, Gallen Triana‐Baltzer, Hartmuth Christian Kolb, Nicholas Ashton, Henrik Zetterberg, Kaj Blennow, Tharick A Pascoal, Maxime Descoteaux, Jesse Klostranec, Pedro Rosa‐Neto

**Affiliations:** ^1^ Translational Neuroimaging Laboratory, The McGill University Research Centre for Studies in Aging, Montréal, QC, Canada; ^2^ Université de Sherbrooke, Sherbrooke, QC, Canada; ^3^ Institute of Neuroscienace and Physiology, University of Gothenburg, Mölndal, Västra Götaland, Sweden; ^4^ Neuroscience Biomarkers, Janssen Research & Development, LLC, San Diego, CA, USA; ^5^ Neuroscience Biomarkers, Johnson & Johnson Innovative Medicine, La Jolla, CA, USA; ^6^ Department of Psychiatry and Neurochemistry, Institute of Neuroscience and Physiology, The Sahlgrenska Academy, University of Gothenburg, Mölndal, Sweden; ^7^ Department of Psychiatry and Neurochemistry, Institute of Neuroscience and Physiology, The Sahlgrenska Academy at the University of Gothenburg, Mölndal, Västra Götalands län, Sweden; ^8^ Institute of Neuroscience and Physiology, Sahlgrenska Academy, University of Gothenburg, Gothenburg, Sweden; ^9^ Departments of Psychiatry and Neurology, University of Pittsburgh School of Medicine, Pittsburgh, PA, USA; ^10^ Division of Diagnostic and Interventional Neuroradiology, Montreal Neurological Institute and Hospital, McGill University Health Centre, Montreal, QC, Canada

## Abstract

**Background:**

It is unclear how cortical microstructure changes across biological stages of Alzheimer's disease (AD), and how this occurs in response to amyloid plaques, tauopathy, and inflammation. We hypothesize that tauopathy is chiefly responsible. To test this, we investigated how different measures of tauopathy, including plasma and PET, influence the microstructure in the whole brain, temporal meta‐ROI, and hippocampus.

**Method:**

We sampled 301 participants (59% female, age 67±10) from the TRIAD cohort with T1 MRI, diffusion‐weighted MRI, amyloid PET (F^18^‐NAV4694), tau PET (F^18^‐MK6240), and plasma biomarkers. We used the NODDI‐flow pipeline to estimate the diffusion characters of the TRIAD cohort (D_parallel_, D_orthogonal_,D_isotropic_) and run the NODDI‐Bingham algorithm to calculate whole‐brain isotropic volume fraction images (“freewater”). FreeSurfer 7 was used to derive hippocampal and temporal meta‐ROI masks (entorhinal, fusiform, interior temporal, and middle temporal gyri). The Standard Uptake Value Ratio (SUVR) method produced amyloid and tau PET images. Plasma biomarkers were measured by Simoa assay. For group comparison, we stratified participants by amyloid SUVR positivity, plasma ptau 217 positivity (T1), and tau SUVR positivity (T2). We performed voxel‐wise correlations between freewater and tauopathy markers via VoxelStats MATLAB toolbox. Mediation analyses were performed in R 4.4.1. Models covaried for amyloid SUVR, age, sex, APOE4 genotype status, and meta‐ROI cortical thickness. Multiple comparisons correction performed by random field theory method.

**Result:**

Tau PET positively correlated with freewater in gray matter within temporal and occipital regions, whereas *p*‐tau217 did so in periventricular white matter (Figure 1A, Figure 2A). The effect of *p*‐tau217 is only present in participants positive for tau tangles (Figure 2C). Freewater increased only in the A+T2+ group in the meta‐ROI and hippocampus (Figure 1B,C). Tau SUVR in meta‐ROI mediated the correlations between both GFAP and ‐tau217 with freewater in the A+T+ group (Figure 3).

**Conclusion:**

In regions of AD‐relevant gray matter, increased freewater correlates with tau tangle accumulation independent of cortical thinning and amyloidopathy. Additionally, the positive correlation of *p*‐tau217 with freewater in the periventricular white matter suggests involvement with ventricular remodeling or local CSF/ISF dynamics. Astrogliosis may be involved with microstructural changes secondary to neurofibrillary tangles.